# Cognition, emotion and reward networks associated with sex differences for romantic appraisals

**DOI:** 10.1038/s41598-018-21079-5

**Published:** 2018-02-12

**Authors:** Jie Yin, Zhiling Zou, Hongwen Song, Zhuo Zhang, Bo Yang, Xiting Huang

**Affiliations:** 1 School of Criminal Justice, China University of Political Science and Law, Beijing, 102249 China; 2 Faculty of Psychology, Southwest University, Chongqing, 400715 China; 3 School of Humanities and Social Science, University of Science and Technology of China, Hefei, 230026 China; 4 School of Socialogy, China University of Political Science and Law, Beijing, 102249 China

## Abstract

Romantic love is a cross-culturally universal phenomenon that serves as a commitment device for motivating pair bonding in human beings. Women and men may experience different feelings when viewing the same warm, romantic scenes. To determine which brain systems may be involved in romance perception and examine possible sex differences, we scanned 16 women and 16 men who were intensely in love, using functional MRI. Participants were required to rate the romance level of 60 pictures showing romantic events that may frequently occur during romantic relationship formation. The results showed that greater brain activation was found for men in the insula, PCC (posterior cingulate cortex), and prefrontal gyrus compared with women, primarily under the High-romance condition. In addition, enhanced functional connectivity between the brain regions involved in the High-romance condition in contrast to the Low-romance condition was only found for men. These data suggest that men and women differ in the processing of romantic information and that it may be  more effortful for men to perceive and evaluate romance degree.

## Introduction

Romantic love is an important and universal human experience^1^. Romantic love has played a critical role in the evolution of *Homo sapiens*. It is a commitment device for motivating pair bonding in humans, enabling them to reproduce and pass on their genes to their children. Only in this way, it is possible for humans to preserve their genes^2^.

Romantic love begins when an individual starts to regard another individual as special and unique^3^. The lover focuses her or his attention on the beloved, overstates the beloved’s virtues and neglects or minimizes her or his flaws, i.e., “love makes blind.” Bartels and Zeki^4^ found that romantic love leads to the suppression of activity in brain regions associated with critical social assessment of other people. This may provide the neurological explanation for the inability of lovers to assess their partners objectively.

In daily life, individuals experience euphoria and well-being when things are going well in a romantic relationship. When things go wrong, individuals undergo a host of negative outcomes including depression, anxiety, and pain^5,6^. As a sophisticated social phenomenon, love influences the behavior, cognition, and emotion of individuals^7^. For example, one study has found that viewing pictures of a romantic partner could reduce pain^8^.

Previous brain imaging research has investigated the neural correlates of romantic love. Bartels and Zeki’s work^9^ was the first fMRI study to explore the neural basis of early-stage romantic love. Along with other studies^10–13^, activation in the dopaminergic reward and motivation system had been observed when participants viewed the photographs of their beloved. The neural correlates of rejection in love have also been investigated^14,15^, which indicated that some individuals who had recently experienced a break-up continued to show activation of the reward system when they thought about or viewed photographs of the person who had rejected them. That is possible because they still want to bond with the beloved and the adversity tends to heighten feelings of romantic love^16^. However, only one neuroimaging study evaluated sex differences in romantic love^17^ and they found the pattern of activation and de-activation was highly similar in men and women.

Some brain imaging research on the cognitive appraisal of social information has considered gender and demonstrated differences between men and women. Harenski *et al*.^18^ found that women and men showed different neural activation when viewing unpleasant pictures and rating the degree of moral violations experienced. Schulte-Rüther *et al*.^19^ showed that the right inferior frontal cortex and superior temporal sulcus were activated more strongly in women than in men, but men showed greater activity in the left temporoparietal junction than women while evaluating their own emotional state as they viewed emotional faces. The authors considered that perhaps the ability to disentangle their own feelings from those observed in other people was greater in men than in women. Furthermore, a study on self- and other-appraisals as well as reflected self-appraisals found that activation in the medial posterior parietal cortex and bilateral temporoparietal junction was greater for men than women under all conditions, while the precuneus showed stronger activation in men than in women specifically during the appraisal of others^20^. Derntl *et al*.^21^ found that women seemed to adopt more emotion- and self-related brain regions, men relied on more cognitive-related areas when solving empathy tasks. These previous studies illustrate that women and men possibly employ divergent brain regions to understand others’ behaviors and emotion.

In the present study, we also recruited participants who were in love and reported intense romantic love for their partners. However, we chose to use images depicting romantic events as stimuli instead of photographs of partners. Participants were instructed to evaluate each picture on its degree of romance while undergoing MRI scanning. The current fMRI study targets the neural differences between men and women in processing romance.

In our last study^22^, we administered short descriptive phrases to participants, and told them to rate the romantic level of the settings depicted by the phrases. Results showed that greater brain activation was found in the frontal lobe, posterior cingulate cortex, and precuneus in men than in women. We did not find any areas related to emotion showing greater activation in women than in men, though Koch *et al*.^23^ suggested that women showed more activation in regions associated with emotional processes when doing social cognition tasks. Since the perception and evaluation of romance involves emotional and cognitive processing, we inferred that the presentation of sentences may not be the most effective way to elicit feelings of romantic love. Therefore, in the present study, we changed the stimulus format to pictures. Holmes *et al*.^24^ has found that imagery evokes stronger affective responses than does verbal processing, perhaps due to the sensitivity of emotional brain regions to imagery. To preserve continuity, we classified the romantic pictures according to their degrees of romance as our previous one^22^. However, in order to simplify the category and data analysis, we divided these items into 2 levels of High- and Low-romance conditions and Medium-romance condition was no longer contained. The first research question in the present study was exploring whether divergent brain regions were activated in appraising romantic pictures of different levels between men and women. Based on previous studies^21^ and our preceding results that sex differences mainly existed in Low-romance level^22^, we expected the brain regions associated with social cognition such as the prefrontal cortex would be more active in men than women, while emotional brain regions such as the posterior cingulate cortex might be more active in women than men, and this activation pattern would mainly appear in low-romance condition.

In addition, we conducted psychophysiological interaction analyses to assess the effect of changes in different romance levels on the interaction between the brain regions involved. Researchers have found that individuals in love showed increased functional connectivity in reward, emotion and social cognition networks compared with the single persons^25^. Additionally, the previous study has found that men activated more areas associated with social cognition and emotion compared with women in romance perception task^22^. Accordingly, we expected functional connectivity in the social cognition and emotion networks to be enhanced mainly in men rather than in women. Moreover, High-romance condition would elicit stronger emotional response than Low-romance condition. Therefore, we hypothesized that the social cognition and emotion networks might be significantly activated during the High-romance condition than during the Low-romance condition in men.

## Results

### Behavioral results

Responses and reaction times (RTs) were recorded. Behavioral data of one female was excluded due to long RTs (>2500 ms). We performed repeated-measures ANOVA with the within-subject factor Condition (high or low romance level) and the between-subject factor Sex.

For reaction times, the ANOVA revealed a main effect of romance level: *F*_(1,39)_ = 31.67, *p* < 0.001, ${\eta }_{p}^{2}$ = 0.45 (*M*_High_ = 1580.21, *SD*_High_ = 309.47; *M*_Low_ = 1714.23, *SD*_Low_ = 292.07), a marginal effect of sex: *F*_(1,39)_ = 3.21, *p* < 0.08, ${\eta }_{p}^{2}$ = 0.08 (*M*_men_ = 1724.41, *SD*_men_ = 320.77; *M*_women_ = 1566.17, *SD*_women_ = 236.10), and a marginal interaction effect between sex and romance conditions: *F*_(1,39)_ = 3.05, *p* < 0.09, ${\eta }_{p}^{2}$ = 0.07. Women responded significantly more quickly to highly romantic stimuli than men did (*t* = 2.16, *p* < 0.05), but there were no sex differences for the low-romance pictures, although the RT was shorter for women than for men.

**Figure 1:**
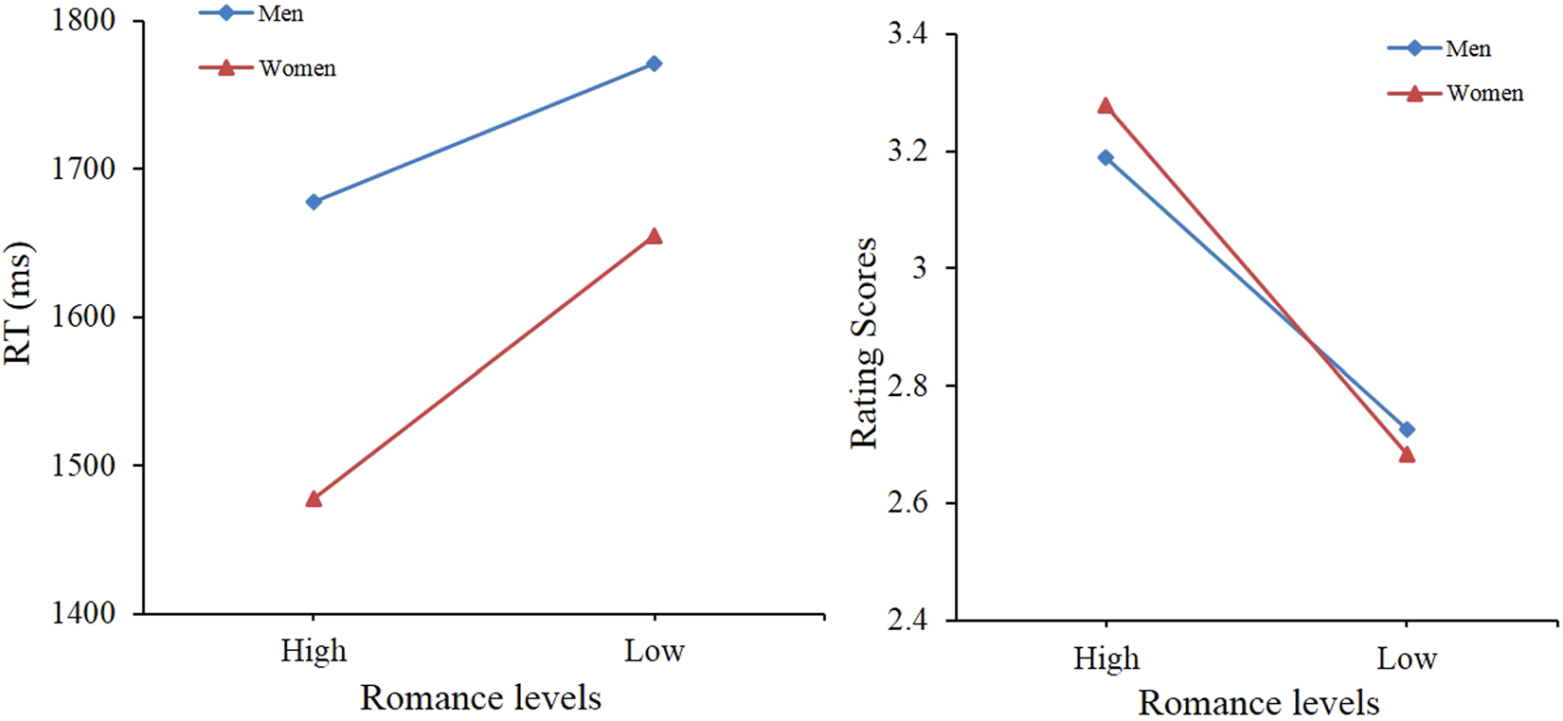
Rating scores and reaction times (RT) for High- and Low-romance conditions.

For rating scores, a main effect of romance level was observed: *F*_(1,39)_ = 136.69, *p* < 0.001, ${\eta }_{p}^{2}$ = 0.78 (*M*_High_ = 3.23, *SD*_High_ = 0.39; *M*_Low_ = 2.70, *SD*_Low_ = 0.40), but no interaction was observed between sex and romance conditions: *F*_(1,39)_ = 2.05, *p* = 0.16. The main effect of sex was not significant: *F*_(1,39)_ = 0.04, *p* = 0.84 (*M*_men_ = 2.96, *SD*_men_ = 0.39; *M*_women_ = 2.98, *SD*_women_ = 0.36). The results are shown in Fig. 1.

### Imaging results

#### Romance levels

**Figure 2:**
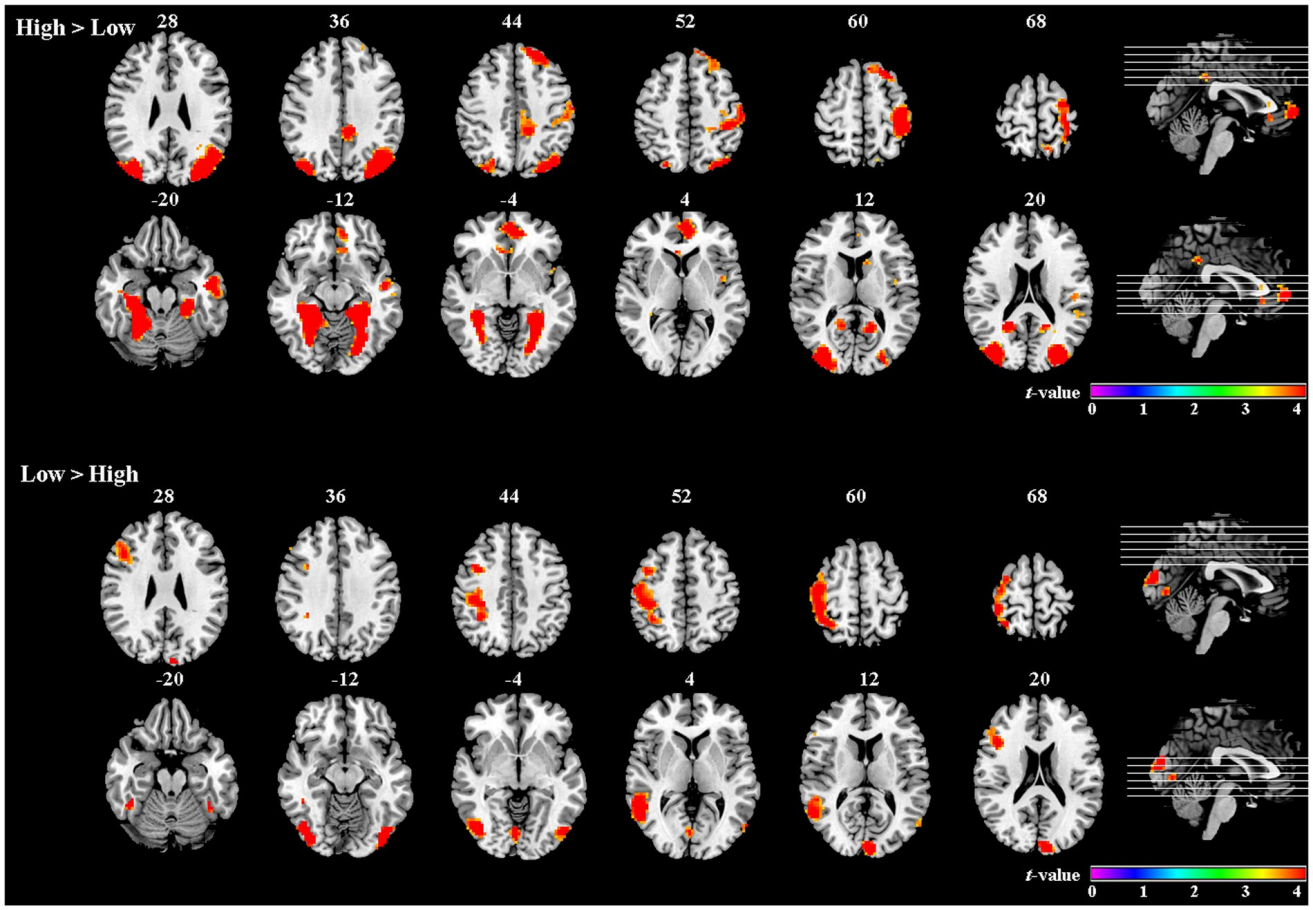
Areas of activation for High- and Low-romance conditions.

The effects of sex and romance level on brain activation were assessed using ANOVA. The main effect of romance level was significant. *Post hoc* tests revealed that the activation of the cingulate gyrus, left caudate, left orbitofrontal cortex, right parahippocampal gyrus, bilateral precuneus, and bilateral fusiform gyri was greater for the High condition than the Low condition. Low-romance items led to stronger activation than High-romance items in the middle and inferior frontal gyri, right precentral and postcentral cortices, and left cuneus. The results are shown in Table 1 and Fig. 2.

**Table 1:** Areas of activation sensitive to romance levels.

Region	Side	BA	voxels	*t-*value	MNI coordinates		
					*x*	*y*	*z*
High > Low							
Medial orbitofrontal cortex	L	10	61	4.69	0	54	−3
Insula	L	13	11	3.82	−42	−3	6
Caudate	L		22	3.86	−12	12	18
Middle temporal gyrus	L	21	131	7.20	−51	−9	−21
Parahippocampal gyrus	R	37	106	9.76	30	−36	−9
Anterior cingulate cortex	L	32	62	4.97	−6	48	0
Middle cingulate cortex	L	31	106	5.39	−12	−42	42
Precuneus	L		26	4.78	−15	−57	15
Precuneus	R		45	5.26	20	−52	18
Fusiform	L	19	231	14.62	−30	−45	−9
Fusiform	R	19	260	9.94	30	−39	−9
Postcentral gyrus	L	3,4	289	5.59	−42	−33	54
Middle occipital gyrus	L	19	310	9.77	−33	−84	27
Middle occipital gyrus	R	19	344	9.92	36	−84	24
Low > High							
Middle frontal gyrus	R	6	66	4.31	36	6	45
Inferior frontal gyrus	R	45	115	4.97	42	21	24
Postcentral gyrus	R	3,40	319	5.27	42	−39	66
Precentral gyrus	R	4	179	4.76	45	−15	57
Middle temporal gyrus	R	22	227	6.54	56	−54	6
Cuneus	L	18	68	6.98	−6	−93	18

#### Sex differences

**Figure 3:**
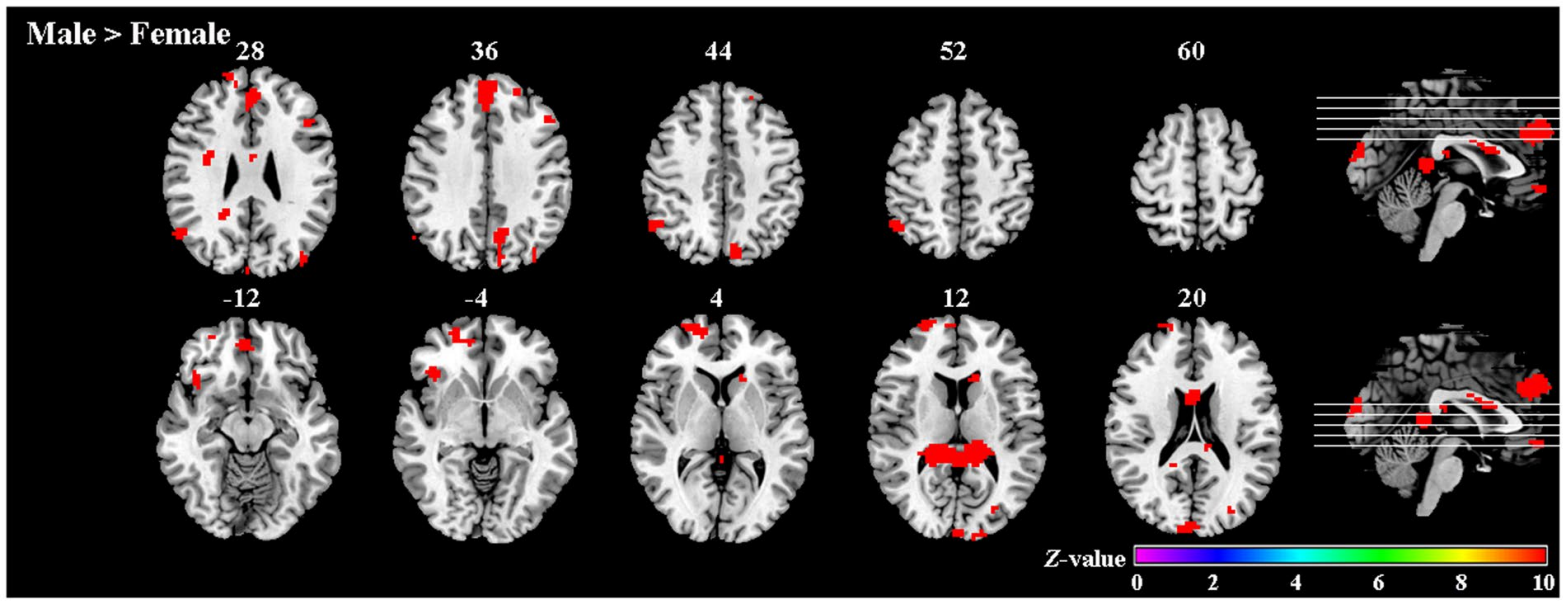
Areas of activation affected by sex differences (Male > Female).

To investigate differences between women and men across romance levels, the main effect of sex was also calculated. As shown in Table 2 and Fig. 3, an effect was demonstrated in the superior frontal cortex, bilateral dorsomedial prefrontal cortices, right medial orbital frontal cortex, bilateral precuneus, left cuneus, right cingulate gyrus, left caudate nucleus, and right insula. *Post hoc* tests revealed that activation of these brain regions was greater for men than women.

**Table 2:** Areas of activation sensitive to sex differences (male > female).

Region	Side	BA	voxels	*t-*value	MNI coordinates		
					*x*	*y*	*z*
Male > Female							
Superior frontal gyrus	L	9	32	24.32	−21	45	39
Superior frontal gyrus	R	10	90	25.61	24	64	6
Superior medial frontal gyrus	L	9	80	23.60	0	51	33
Superior medial frontal gyrus	R	10	69	20.46	12	60	3
Medial orbitofrontal gyrus	R	11	36	17.68	4	48	−9
Precuneus	L	7	57	25.84	−9	−75	42
Precuneus	R	7	9	14.46	18	−57	33
Cuneus	L	18	26	29.39	0	−96	15
Insula	R	47	26	26.66	39	18	−9
Middle cingulate cortex	R		12	17.38	3	36	30
Posterior cingulate cortex	R		33	31.79	12	−39	12
Caudate	L		13	17.84	−15	24	6
Middle occipital gyrus	L	19	21	31.92	−27	−81	18

Simple effect analysis was performed to investigate differences between women and men for each romance condition. Men activated more brain regions than women for the High-romance condition. These regions were localized in the right insula, right superior frontal gyrus, right posterior cingulate cortex. For the Low-romance condition, men showed greater activation than women mainly in the right posterior cingulate cortex. At the chosen threshold criteria, no regions were activated more strongly in women than in men.

#### Psychophysiological interaction analysis

The insula and PCC were observed activation in previous studies on romantic love^4,10,11,14,25^, which were involved in emotion regulation^26^. The mPFC has been found to play a role in social cognition^27^. Based on these previous results, we selected four regions of interest (ROIs) as seed regions for the PPI analysis (see Table 3): the right posterior cingulate cortex (PCC_R), the right insula (Insula_R), the left medial prefrontal cortex (mPFC_L), and the right medial prefrontal cortex (mPFC_R). These brain regions of the same ROI in different literatures were made to small 10 mm radius spheres on the basis of the peak coordinate in these previous results. Then image calculation was performed to obtain the overlap of these spheres. Moreover, to ensure that the resulting overlaps included only voxels of one brain region, these overlaps were additionally masked with a corresponding region-mask to exclude neighboring anatomical structures.

**Table 3:** Seed regions of interest (ROIs) and their MNI coordinates.

ROI	side	MNI		
		*x*	*y*	*z*
PCC	R	4	−42	10
mPFC	L	0	51	33
mPFC	R	6	48	30
Insula	R	39	18	−9

**Figure 4:**
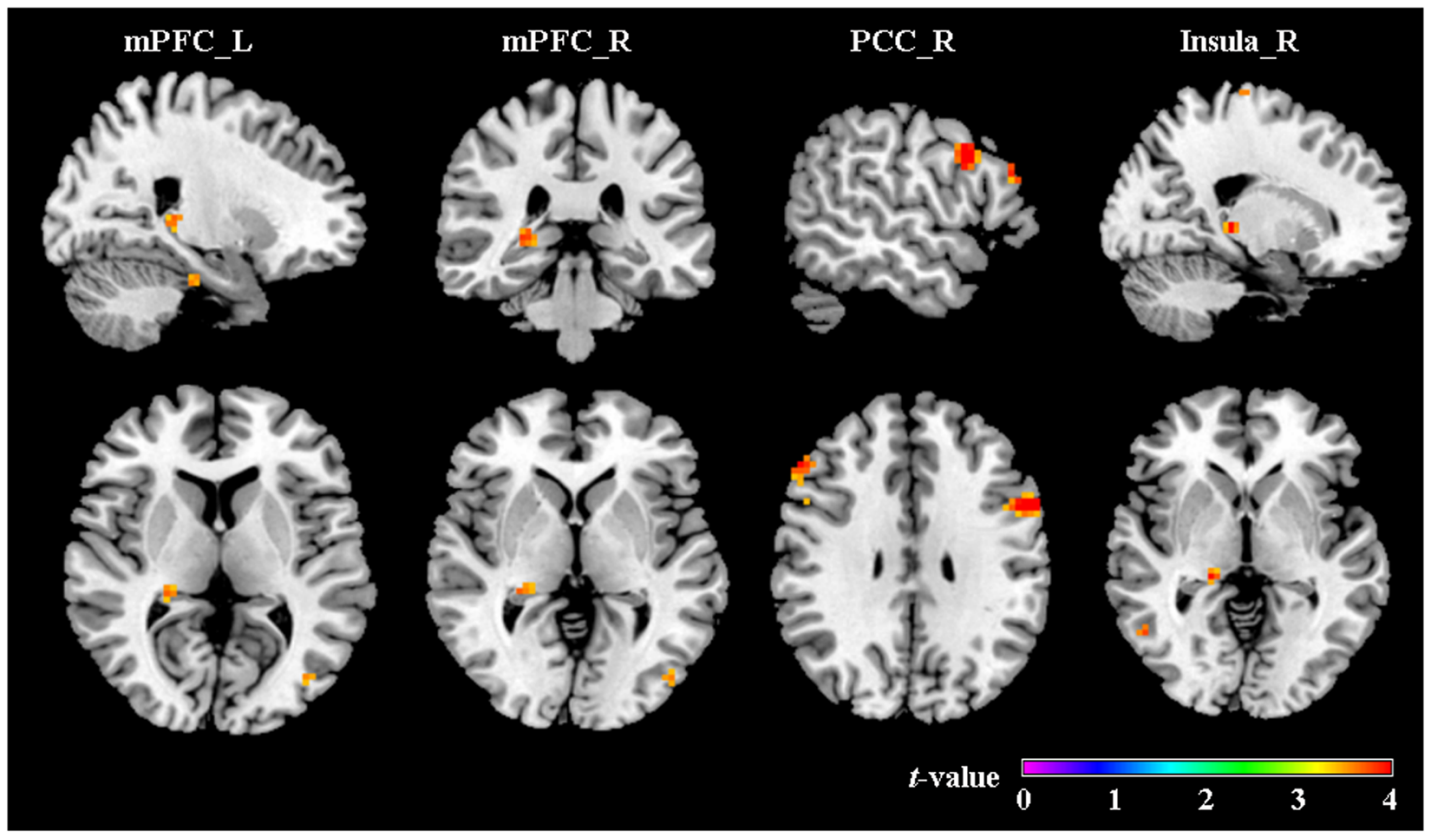
Significant regions in the comparison of functional connectivity between Low and High condition in males.

The between-group comparison results from the PPI analysis showed that in men compared with that in women, the activity in the right posterior cingulate cortex (rPCC) was accompanied by an increased functional interaction with the bilateral inferior frontal gyri, right middle and medial frontal gyri, and right calcarine cortex from the Low to the High condition. The left mPFC showed a greater correlation with the right inferior and middle temporal gyri, right hippocampus, and left anterior and middle cingulate cortex during the High condition than during the Low condition. A greater connectivity of the right mPFC was observed with the right hippocampus, right middle and inferior temporal cortex and left middle occipital cortex during the High condition than during the Low condition. The right insula showed a greater correlation with the right middle frontal gyrus, bilateral anterior cingulate cortex (ACC), left precuneus, right inferior and middle temporal gyrus in the High condition than the Low condition. At the same threshold, there was no significant functional interaction from Low to High in women compared with that in men, or from High to Low in either sex group (see Table 4; Fig. 4).

**Table 4:** Significant functional interaction from Low- to High-romance condition.

Seed ROI	location of region	Side	voxels	*t*-value	MNI coordinates		
					x	y	z
Male > female							
PCC_R							
	Triangle inferior frontal gyrus	L	48	3.90	−54	30	24
	Triangle inferior frontal gyrus	R	62	4.02	54	30	30
	Supperior medial frontal gyrus	R	18	3.32	12	39	39
	Middle frontal gyrus	R	136	4.15	30	21	57
	Calcarine cortex	R	65	3.79	9	−69	9
mPFC_L							
	Inferior temporal gyrus	R	69	3.95	51	−72	−9
	Middle temporal gyrus	R	81	3.61	51	−63	0
	Middle occipital gyrus	L	102	3.57	−39	−75	6
	Hippocampus	R	28	3.70	24	−33	6
	Anterior cingulate cortex	L	20	3.43	−3	6	30
	Middle cingulate cortex	L	19	3.12	0	−6	33
mPFC_R							
	Hippocampus	R	37	3.83	27	−33	3
	Middle occipital cortex	L	66	3.80	−45	−78	0
	Inferior temporal gyrus	R	60	3.84	51	−72	−9
	Middle temporal gyrus	R	48	3.42	51	−63	0
Insula_R							
	Middle frontal gyrus	R	17	3.49	24	42	24
	Anterior cingulate cortex	L	39	3.57	0	27	24
	Anterior cingulate cortex	R	26	3.59	3	24	24
	Middle cingulate cortex	R	52	3.37	2	24	30
	Precuneus	L	21	4.76	−6	−81	45
	Inferior temporal gyrus	R	106	4.07	54	−51	−15
	Middle temporal gyrus	R	27	3.78	51	−60	0
	Inferior parietal gyrus	R	173	4.51	30	−48	51
	Middle occipital gyrus	L	41	3.45	−48	−78	0
Female > Male							
	none

## Discussion

The current fMRI study investigated romantic appraisals in male and female Chinese college students. The High-romance condition activated more brain regions in the prefrontal cortex, cingulate cortex, precuneus and caudate relative to the Low-romance condition. Highly romantic images seem to be idealized scenes that can elicit stronger emotional responses than stimuli that are not as romantic. The areas associated with emotion (the cingulate cortex and insula) were preferentially involved in High-romance condition compared with Low-romance condition. The caudate nucleus, a key area of the brain’s dopaminergic reward system^28^, was also found to be activated in previous studies on love^4,10,12–14^. The parahippocampal gyrus and middle temporal gyrus have been found implicated in memory, episodic memory^29,30^. The left postcentral gyrus was also found to be activated in our previous study on romance perception^22^.

The Low-romance condition showed a large amount of activation in the right postcentral, precentral gyrus, frontal lobe, and temporal lobe, as well as part of cuneus compared with the High-romance condition. Coupled with the behavioral results from the rating scores, low-level romantic stimuli were perceived weakly, and individuals’ feelings were not as strong as those evoked by highly romantic stimuli. The present imaging results for romance levels were similar to the previous study that using romantic sentences^22^. This once again illustrated that low-level romantic stimuli might require more cognitive processing compared with high-level stimuli.

Considering the effects of sex, the behavioral data revealed no differences in the rating scores between men and women for High or Low condition. However, the imaging results showed that over all romance conditions, men and women displayed significantly different activation patterns when performing the evaluation task. The male minus female comparison showed activation in the frontal lobe (e.g., the bilateral mPFC, superior frontal cortex, and right OFC), right middle cingulate and posterior cingulate cortex, bilateral precuneus, left caudate nucleus, and right insula, as well as the occipital lobe.

The dorsomedial prefrontal cortex (dmPFC) has been linked to social cognition, e.g., mentalizing, which describes overt thoughts about the internal mental states of others^27,31^. The precuneus was also found to be activated in our last study^22^. Some functional imaging findings suggest that the precuneus (BA 7) is associated with visuo-spatial imagery^32,33^. However, an alternative possibility is that increased activity in the precuneus does not specifically represent visuo-spatial processing, but may represent self-related processing, such as self-referential judgements from a first- versus third-person perspective^31^. In addition, the precuneus has been implicated more in understanding others compared with the Self ^20,34^. These results confirmed previous research which showed that males perform worse on mentalizing about emotions and actions of self and others than females^35^, so men recruited these regions more.

More activation for males than females was also found in PCC, which was associated with emotion^36^, especially sensitive to pleasant visual stimuli^37^. It was activated more in responses to images of lovers than of highly familiar acquaintances^38^.

The subcortical regions (the insula and caudate nucleus) also showed greater activity in men relative to women. The insula, which has been related to emotion regulation and awareness of sensation^39,40^, also plays a role in reward and addiction^41,42^. The caudate has been involved in reward anticipation and motivation^43,44^. However, an alternative interpretation for its greater activation in men may be that men to some degree regard the romantic/cozy scenes as a cue for sexual intercourse, since some other studies have found that the caudate was associated with sexual arousal^45,46^, whereas this is not the case for women.

In addition, we also conducted simple effect analysis. The results showed that large sex differences appeared mainly in the High-romance condition. These regions included the right insula, right posterior cingulate cortex and right superior frontal gyrus. In the Low-romance condition, more activation for men than women was only found in the right posterior cingulate cortex.

These results indicate that while women and men show similar behavioral evaluations of romantic stimuli, they display a different neural activation pattern in the brain when generating these evaluations. There are two possible explanations. First, probably the sample size was too small to reach significance in the behavioral test. Second, at behavioral level men and women were likely to perceive the same degree of romance when exposed to these images, but they may recruit different cognitive processing strategies in their brains to obtain the similar evaluation. The imaging results for the effect of sex in the present study using pictures were similar to the effect of sex observed in our previous study using romantic sentences^22^, i.e., men activated more brain regions than did women, whereas women did not show any areas with greater activation than men at the chosen threshold. This brain activation pattern associated with sex also appeared in the study of social appraisals^20^. It seems that men rely to a greater extent on a neural mechanism for processing social information.

The differences between the present study and our previous study were that sex differences in the present study were found primarily at high romance level, whereas in our previous study this effect mainly appeared at low romance level. The activation differences between the two studies may result from differences between words and pictures. When viewing the phrases, individuals might imagine the romantic scenes. They didn’t have to do that when viewing the pictures. To sum up, the brain regions with greater activation in men compared with women were primarily associated with social cognition, emotion, and reward. We have still not found stronger activation in brain regions related to emotion in women compared with men. Therefore, the present results just supported our first hypothesis partially.

To further assess the relationship between these related regions during the Low and High evaluation tasks in men and women, a PPI analysis was performed to examine the functional integration of romantic appraisals. The mPFC showed an increased functional interaction with the temporal cortex, hippocampus, and cingulate cortex from the Low to the High condition in men compared with that in women. It seems that when perceiving and evaluating highly romantic stimuli, the connectivity of the brain regions associated with visual perception, memory, and social evaluation is enhanced. The PCC showed stronger correlations during the High condition than the Low condition with the frontal cortex (inferior, medial and middle frontal gyri) in men relative to women, which might indicate that the connectivity of evaluation - emotion network was enhanced in High-romance condition in men.

The insula showed an increased functional link with the cingulate cortex, precuneus, and temporal gyrus from the Low to the High condition in men compared to women, which suggests that the cognitive evaluation-reward network may play an important role in the High condition. These results also indicate that highly romantic stimuli can not only elicit stronger emotional responses and rewards than less romantic stimuli, but the functional connectivity between these regions was also greater for High relative to Low romantic stimuli. However, this connectivity pattern was observed mainly in men, but not in women. The results of the PPI analyses supported our second hypothesis. For men, the High-romance condition showed higher functional interaction in social cognition and emotion networks than did the Low-romance condition.

In conclusion, the study revealed that men relied on more brain regions to perceive and evaluate these pictures, and had stronger functional connectivity between related regions relative to women. These results suggest that more effort is needed for men to perform the romance evaluation task. That is, it’s harder for men to recognize romantic situations and judge the degree of romance. Their longer RTs also illustrated this. Somewhat consistent with previous findings^18^, men rely heavily on analytical thinking and employ justice-based evaluation, while women are more sentimental and employ care-based evaluation. Men are more likely to start a relationship and terminate it. From an evolutionary point of view, it is conducive for them to conserve energy to choose a suitable partner, thereby facilitating gene preservation. It becomes clearer why men are more initiative in a relationship.

There were some limitations in the present study, for example, the lack of a control condition of evaluating other dimension (not just the degree of romance). Therefore, the present sex differences seem to a certain degree reflecting that men think harder in classifying than women and are not particularly specific to romance evaluation. Besides, neutral stimuli were not included in the study. The results might be more explicit if a comparison of the romance condition and the control condition was made. In addition, the present study included only the in-love group. Whether the gender effect also applies to the not-in-love group would be tested and verified in future research.

There is an issue we may also need to note, that is, Collectivistic (Eastern) and individualistic (Western) cultures show differences in many respects, for example, attitudes to love and marriage^47^. Yet, the fact that the study from the Netherlands by Veroude *et al*.^20^ showing a similar activation pattern as our previous study^22^ between men and women in social appraisals suggests that people from Eastern and Western cultures may share some common neural mechanisms in social-related processing. What’s more, Xu *et al*.^13^ used the same procedure as Aron *et al*.^10^ to study Chinese participants and found that the brain regions activated were similar to other westerners’ studies^9,10^. Therefore, the present results may to some extent be representative. Further studies should be carried out to confirm this.

## Conclusion

In the present study, we demonstrated important differences between men and women in the neural bases of romantic evaluation. Men activated more brain regions than did women in the PCC, insula and prefrontal gyrus, and enhanced functional connectivity between the brain regions was observed from the Low to the High condition in men, but not in women. This study provides another empirical evidence on romance perception, verifies and supplements the previous results on sex differences.

## Methods

### Participants

Forty-two, right-handed adults were recruited (21 women, aged 20.81 ± 2.27 years; 21 men, aged 21.19 ± 2.29 years) from the Southwest University (SWU) through an advertisement on the university bulletin board system. All were in an intense love relationship scoring high on the Passionate Love Scale (PLS)^48^ (*M* = 4.43, *SD* = 0.61), and men and women didn’t show significant differences on PLS: *t* = 1.16, *p* > 0.05 (*M*_men_ = 4.54*, SD*_men_ = 0.64; *M*_women_ = 4.32*, SD*_women_ = 0.57). For this sample, the duration of the romantic relationship had ranged from 3 to 18 months (*M* = 8.76, *SD* = 5.42). All of the lovers were at SWU, so they could connect with each other frequently. The mean length of communication was 3.98 h per day. The two sex groups were matched for the level of education. Five male participants and five female participants were excluded due to excessive head motion during scanning (>2 mm), leaving 16 men and 16 women in the final fMRI analysis. None of them reported any neurological or psychiatric disorders. Pre-screening interviews were conducted to verify that they were heterosexual (self-reported as having only opposite-sex sexual desire and partners). All participants provided written informed consent and were paid 50 RMB as compensation for their time. The Ethics Committee at Southwest University approved the study and the methods were carried out in accordance with the relevant guidelines and regulation.

### Stimuli and procedure

We collected 110 pictures of scenes in which lovers were together, mostly obtained from the internet. Some of them seemed to be highly romantic, for example, lovers sitting on a beach with a sunset; the others seem to be slightly romantic, for example, lovers shopping in a supermarket. All the pictures were clipped to the same size using Adobe Photoshop (Adobe Systems Inc., San Jose, CA, USA). We then recruited 240 college students from SWU to assess the occurrence frequency, sexual arousal, degree of happiness, and degree of romance associated with the events depicted in the images. Each dimension was assessed from 0 to 9 (0 = *not at all*, 9 = *extremely*). Finally, 60 pictures were selected based on their ratings. Among them were 30 pictures that were higher in their degree of romance (*M* = 6.77, *SD* = 0.538), and these were named the High-romance condition. The other 30 pictures were lower in their degree of romance (*M* = 5.091, *SD* = 0.559), and were classified as the Low-romance condition. The differences between the two conditions in the four dimensions are shown in Table 5.

**Table 5:** The differences between High and Low conditions in the four dimensions.

dimension	*Mean* (SD)		*t*	${\eta }_{p}^{2}$
	High	Low		
Degree of happiness	7.25(0.44)	6.27(0.52)	7.84**	0.69
Sexual arousal	2.87(0.60)	2.70(0.67)	1.04	—
Occurrence frequency	4.84(1.29)	4.58(1.10)	0.83	—
Degree of romance	6.77(0.54)	5.09(0.56)	11.84**	0.82

**Indicates *p* < 0.001.

The 60 images were presented on a silver background using E-Prime software (Psychology Software Tools, Inc. Pittsburgh, PA, USA) in two runs, each lasting approximately 10 min. Each picture was 640 × 480 pixels. Participants pressed buttons to indicate their evaluation of the degree of romance associated with each image. Responses were recorded using a fiber-optic finger-switch response system.

Each participant completed two runs. In each run, all 60 pictures (30 for each type: high and low romance levels) were presented in a pseudo-random order. Run 2 was a repeat of run 1 (with a different stimulus-presentation order) to obtain more data points. For each run, there was a 16-s fixation prior to the first item and a 10-s fixation after the last one. Each picture was presented for 4 s, during which the participant evaluated the degree of romance in the picture. Next, a variable blank screen (2, 4, or 6 s) was shown, followed by a 2-s fixation. The use of variable duration is a common practice in event-related fMRI designs to enhance the power of the regression analysis and to reduce the participants’ anticipation of incoming stimuli. The two types of pictures (high or low romance level) were in pseudo-random order.

Before entering the scanner, each participant was asked about the commencement, duration, intensity, and feelings of their love relationship. They also completed the PLS to test the intensity of passion in their relationship. They then needed to complete a practice procedure to ensure that they understood how to perform the task. The practice procedure was the same as the experimental procedure, but with only five trials. Participants were instructed to evaluate the degree of romance for each picture via a button press with four keys (1 to 4; 1 = *not at all romantic*, 2 = *slightly romantic*, 3 = *quite romantic*, 4 = *extremely romantic*) when viewing the pictures on the screen.

Following scanning, we conducted exit interviews to determine whether the participants had followed the instructions and assess their feelings during the fMRI scan. All participants reported performing reasonably well, with little head movement.

### MRI data acquisition

Whole-brain imaging data were obtained using a Siemens 3-T Marconi Edge MRI system (Siemens AG, Germany). Blood-oxygen-level-dependent (BOLD) responses and in-plane anatomical data were recorded for each participant. A T2*-weighted echo planar imaging sequence was used with the following parameters: time to repetition (TR) = 2000 ms, time to echo (TE) = 30 ms, flip angle (FA) = 90°, field of view (FOV) = 22 × 22 cm, slice thickness = 3 mm, matrix size = 64 × 64, number of slices = 32, Voxel size = 3.4 × 3.4 × 3.0 mm. A T1-weighted anatomical scan was also acquired to aid with spatial normalization (TR = 1900 ms, TE = 2.52, FA = 9°, FOV = 25 × 25 cm, slice thickness = 1 mm, matrix size = 256 × 256, number of slices = 32, Voxel size = 1.0 × 1.0 × 1.0 mm).

### fMRI data analyses

The fMRI data were analyzed using Statistical Parametric Mapping (SPM8, www.fil.ion.ucl.ac.uk/spm). First, the data for all participants were preprocessed. Images were realigned using a six-parameter rigid-body transformation to correct for head movement. The functional images were coregistered to the structural image and normalized to the MNI template. Spatial smoothing was performed with an 8-mm full width at half maximum isotropic Gaussian kernel.

The general linear model was applied to estimate the effects of High *versus* Low romance conditions for each participant. In a first-level analysis, each event was specified individually. Each trial (pictures with high or low levels of romance) was specified as a separate regressor and convolved with the canonical hemodynamic response function. High-pass filtering was used to remove low-frequency noise. Motion parameters were included as regressors of no interest. The resulting parameter estimates for the Low < the High contrast for each participant was entered into a second-level analysis.

An ANOVA was conducted with romance level as a within-subject factor (High, Low) and sex as a between-subject factor (female, male). To define the effects, pairwise comparisons were conducted between different romance conditions, as well as the two sex groups. AlphaSim corrected *p* threshold of 0.05 (combination of voxel level 0.001 uncorrected and cluster size 22 voxels determined by a Monte Carlo simulation) was chosen for all contrasts.

### Psychophysiological interaction (PPI) analysis

To assess the hypothesis that men might show increased functional connectivity in the social cognition network and emotion network than women during the High-romance condition compared with Low-romance condition, we estimated the functional interaction of the brain regions involved using the PPI analysis methodology implemented in SPM8. PPI detects task-specific increases in the relationship between a seed region of interest and the rest regions of the brain, measured in terms of the strength of the regression of activity in one region on another^49^. It consists of a design matrix with three regressors: (i) the “psychological variable,” representing the cognitive processes (here High-romance condition *vs*. Low-romance condition), (ii) the “physiological variable,” representing neural responses in the designated brain regions, and (iii) the interaction term of (i) and (ii). First, the deconvolved time course was extracted from these seed regions of interest, reflecting sex differences in perceiving and evaluating romance. Then the time course for each seed region was compared between High-romance condition and Low-romance condition in the PPI analysis, resulting in contrast images for each region of interest for each subject. The contrast images were then entered into second-level group analyses using a two-sample *t*-test for each seed region to test sex effect (AlphaSim corrected, *p* < 0.05. Combination of voxel level *p* < 0.005 and cluster size >46 voxels, determined by a Monte Carlo simulation).

### Data availability

The authors declare that the data of the study is available.
